# The Best Treatment for the Umbilical Cord

**Published:** 1893-12

**Authors:** C. D. Hurt

**Affiliations:** Atlanta, Ga.


### THE BEST TREATMENT FOR THE UMBILICAL CORD.

BY C. D. HURT, M. D.,
ATLANTA, GA.

In a review from your August number I notice an article with the above heading.

It is desirable in the treatment of the cord stump to avoid both the risk of infection by the absorption of putrid matter, and the unpleasant putrefactive odor which is sometimes generated.

At the same time it is unnecessary and undesirable to have the cord continue intact and undetached for a protracted period of time under the use of antiseptics. After birth the functions of the cord are no longer needed and its stump is a useless appendage which nature seeks to remove.

From our observation of the new born quadruped we learn the value of safe antiseptics provided in nature. The animal lies upon the ground while the moisture of the cord causes the adhesion to it of dust or pulverized earth sufficient to serve as a drying absorbent and antiseptic.

A good dressing for the cord of an infant is composed of equal parts of aristol, lapis caliminaris and subnit. bismuth, applied until no moisture shows through the powder. Then wrap in soft linen or absorbent cotton, apply the binder and leave undisturbed until the fourth or fifth day, when usually it is found detached. If any irritation remains after the cord drops away dust it with the same powder and it will heal very kindly.

The irritating properties of glycerine to the tender cuticle of a new born babe sometimes cause pain and discomfort, while the powder is never unpleasant.

